# Primary care providers and hypertension in pregnancy: Reflections on a patient encounter

**DOI:** 10.4102/safp.v62i1.5086

**Published:** 2020-07-13

**Authors:** Jagidesa Moodley, Prakash Jugnanden, Mergan Naidoo, Nnabuike C. Ngene

**Affiliations:** 1 Department of Obstetrics and Gynaecology, School of Clinical Medicine, College of Health Sciences, University of KwaZulu-Natal, Durban, South Africa; 2 Private Practice, Durban, South Africa; 3 Department of Family Medicine, Faculty of Health Sciences, University of KwaZulu-Natal, Durban, South Africa; 4 Department of Obstetrics and Gynaecology, School of Clinical Medicine, Faculty of Health Sciences, University of the Witwatersrand, Johannesburg, South Africa; 5 Department of Obstetrics and Gynaecology, Klerksdorp Hospital, Klerksdorp, South Africa

**Keywords:** immediate lowering of acute severe hypertension, severe pre-eclampsia, triage, education, family medicine

## Abstract

In South Africa, large numbers of individuals with medical emergencies initially visit a general practitioner or family physician. In the case of maternity care, this may occasionally involve acute onset of severe hypertension during the antenatal period. Primary care providers (PCPs) are therefore faced with the conundrum of treating and stabilising high blood pressure or referring the patient to an appropriate hospital. Case reviews within groups of medical practitioners provide an opportunity for learning in the practical management of obstetric emergencies. The case history of a patient with severe hypertension was presented, and reflections on this patient encounter were highlighted. Amongst the challenges faced by generalists in their interactions with the public health sector are availability of standard clinical protocols, medicines, the need to work in partnership and the need to have ‘feedback’.

## Introduction

In South Africa, severe hypertension in pregnancy (SHP) is the most common direct cause of maternal mortality.^1^ Many pregnant women from the low socio-economic strata seek medical advice and help from private medical practitioners, including family physicians in general practice, particularly if they have severe symptoms. This health-seeking behaviour may be the result of anxiety of long waiting periods, poor staff attitudes and the possibility of being turned away from public health facilities for various reasons.^2^ Private primary care providers (PCPs) should therefore be prepared to deal with obstetric emergencies although they may only rarely encounter such situations. A recent case of a young pregnant woman presenting to a PCP indicated a ‘red flag’ in obstetric parlance and created a dilemma on whether to initiate emergency treatment or immediately refer the patient without intervention. The patient encounter presented below explored the clinical and contextual conundrums and possible lessons to learn, particularly in view of the introduction of the national health insurance (NHI) system in South Africa.

## The patient’s clinical summary

A 26-year-old, primigravida presented to a PCP with a 3-day history of constant abdominal pain and 1-day duration of persistent headache. In addition, the patient had had two ‘normal’ antenatal care visits at a district hospital. The patient had her last antenatal clinic visit 3 weeks earlier. The patient was 28 weeks pregnant.

On physical examination, the patient had a pulse rate of 84 beats/min and blood pressure (BP) of 182/104 mmHg. The chest was clinically ‘clear’, the abdomen was soft, but there was right upper quadrant abdominal tenderness. Abdominal palpation revealed the evidence of a pregnancy of 28 weeks gestational age with the foetal heart present. The PCP elicited brisk reflexes and 3+ proteinuria was measured using a urinary dipstick.

The PCP made a diagnosis of imminent eclampsia and decided to transfer the patient immediately to a nearby district hospital.

As the PCP recognised this to be an obstetric emergency, following shared decision-making with the patient of the likely diagnosis (imminent eclampsia), and because of the fact that she was accompanied by a relative, who had a motor vehicle, immediate referral to the district hospital was made.

### Ethical consideration

This is a case report, and there were no identifiers used of patients, families and health facilities.

## Discussion

### Reflections on the patient encounter

The patient had SHP. Although this is a rare event, particularly for private primary healthcare practitioners, it is important to recognise that cerebral haemorrhage is the most common cause of mortality associated with SHP.^1^ The Maternity Care Guidelines for clinics and district hospitals recommend immediate but judicious lowering of severe hypertension (BP ≥ 160 mmHg systolic and/or diastolic ≥ 110 mmHg).^3^ The standard clinical guidelines also recommend the use of nifedipine (10 mg tablet) orally; this calcium channel blocker lowers the high BP within 20–30 min.^3,4^ Nifedipine tablets therefore should be kept in the healthcare emergency trolley of all primary healthcare practitioners. In this patient encounter, the pregnant woman had symptoms and signs associated with severe hypertension. Headache, nausea and vomiting, visual disturbances and the presence of brisk references are suggestive of imminent eclampsia. The maternity care guidelines for district hospitals and clinics in South Africa recommend the use of magnesium sulphate for the prevention of seizures and eclampsia in such circumstances.^3,5^

Other antihypertensive agents commonly used in pregnancy in South Africa are alpha methyldopa (Aldomet) and intravenous labetalol.^3^ Aldomet has a variable onset of action and is not usually used for SHP, although it may be used in combination with a rapid-acting antihypertensive agent.^3^ Labetalol is used intravenously, has a slow bolus infusion and has a similar onset of action as nifedipine.

The important principle in the management of SHP is to lower high BP as quickly as possible so as to ‘stabilise’ the hypertensive disorder.^4^ The clinical guidelines in the public sector suggest that in women with imminent eclampsia (hypertension with symptoms and signs such as headache and epigastric pain), magnesium sulphate can be used to prevent convulsions.^5^

Therefore, general practitioners should keep magnesium sulphate in their emergency trolleys in the event of encountering a patient with imminent eclampsia. A recent publication on an approach to hypertensive disorders during pregnancy for the primary care physician recommends ‘stabilising’ the patient (lowering severe high BP), informing the receiving hospital, giving magnesium sulphate if the receiving doctor suggests it and facilitating referral on the same day.^5^ Certainly, on reflection, this patient encounter highlights the challenges of contacting a referral hospital, obtaining an emergency ambulance and providing feedback on the case to the primary healthcare professional. Therefore, there should be discussions between primary healthcare professional organisations and the public health district healthcare offices to ensure safe health services for pregnant women. Furthermore, challenges faced by primary healthcare professionals in private practice include:

- the availability of standard clinical protocols for managing obstetric emergencies because the patient eventually links to public health hospitals
- the availability of medicine used in the management of obstetric emergencies
- the need to establish a network of healthcare professionals at a district healthcare level, which includes private healthcare practitioners and practitioners in public healthcare facilities, to ensure shared clinical protocols, referral indicators and the pathways
- to ensure a system of ‘feedback’ to primary healthcare providers to ensure that the PCP is informed of the patient outcome.

In summary, PCPs must be aware that any pregnant woman who has right upper quadrant abdominal pain that radiates to the shoulder, hypertension and severe headache warrants immediate lowering of BP using a quick-acting antihypertensive agent, stabilisation of BP and referral to the appropriate level of care. The district health authority and the academic institutions have an important role in continuing medical education for PCPs.

Finally, PCPs can play an important role in continuing the management of patients with pre-eclampsia, because such patients may have elevated BP in the puerperium and may need to continue their antihypertensive medications; some are likely to develop cardiovascular problems later in life and therefore need at least yearly medical reviews to ensure that they have not developed chronic hypertension, cardiomyopathy and/or diabetes mellitus.
